# Artificial Intelligence Based Patient-Specific Preoperative Planning Algorithm for Total Knee Arthroplasty

**DOI:** 10.3389/frobt.2022.840282

**Published:** 2022-03-08

**Authors:** Adriaan Lambrechts, Roel Wirix-Speetjens, Frederik Maes, Sabine Van Huffel

**Affiliations:** ^1^ Materialise NV, Leuven, Belgium; ^2^ Department of Electrical Engineering (ESAT), STADIUS Center for Dynamical Systems, Signal Processing and Data Analytics, KU Leuven, Leuven, Belgium; ^3^ Department of Electrical Engineering (ESAT), Processing Speech and Images (PSI), KU Leuven, Leuven, Belgium; ^4^ Medical Imaging Research Center, UZ Leuven, Leuven, Belgium

**Keywords:** total knee arthroplasty, patient-specific, preoperative planning, machine learning, orthopedic surgery, support vector machine, artificial intelligence

## Abstract

Previous studies have shown that the manufacturer’s default preoperative plans for total knee arthroplasty with patient-specific guides require frequent, time-consuming changes by the surgeon. Currently, no research has been done on predicting preoperative plans for orthopedic surgery using machine learning. Therefore, this study aims to evaluate whether artificial intelligence (AI) driven planning tools can create surgeon and patient-specific preoperative plans that require fewer changes by the surgeon. A dataset of 5409 preoperative plans, including the manufacturer’s default and the plans corrected by 39 surgeons, was collected. Features were extracted from the preoperative plans that describe the implant sizes, position, and orientation in a surgeon- and patient-specific manner. Based on these features, non-linear regression models were employed to predict the surgeon’s corrected preoperative plan. The average number of corrections a surgeon has to make to the preoperative plan generated using AI was reduced by 39.7% compared to the manufacturer’s default plan. The femoral and tibial implant size in the manufacturer’s plan was correct in 68.4% and 73.1% of the cases, respectively, while the AI-based plan was correct in 82.2% and 85.0% of the cases, respectively, compared to the surgeon approved plan. Our method successfully demonstrated the use of machine learning to create preoperative plans in a surgeon- and patient-specific manner for total knee arthroplasty.

## 1 Introduction

**FIGURE 1:**
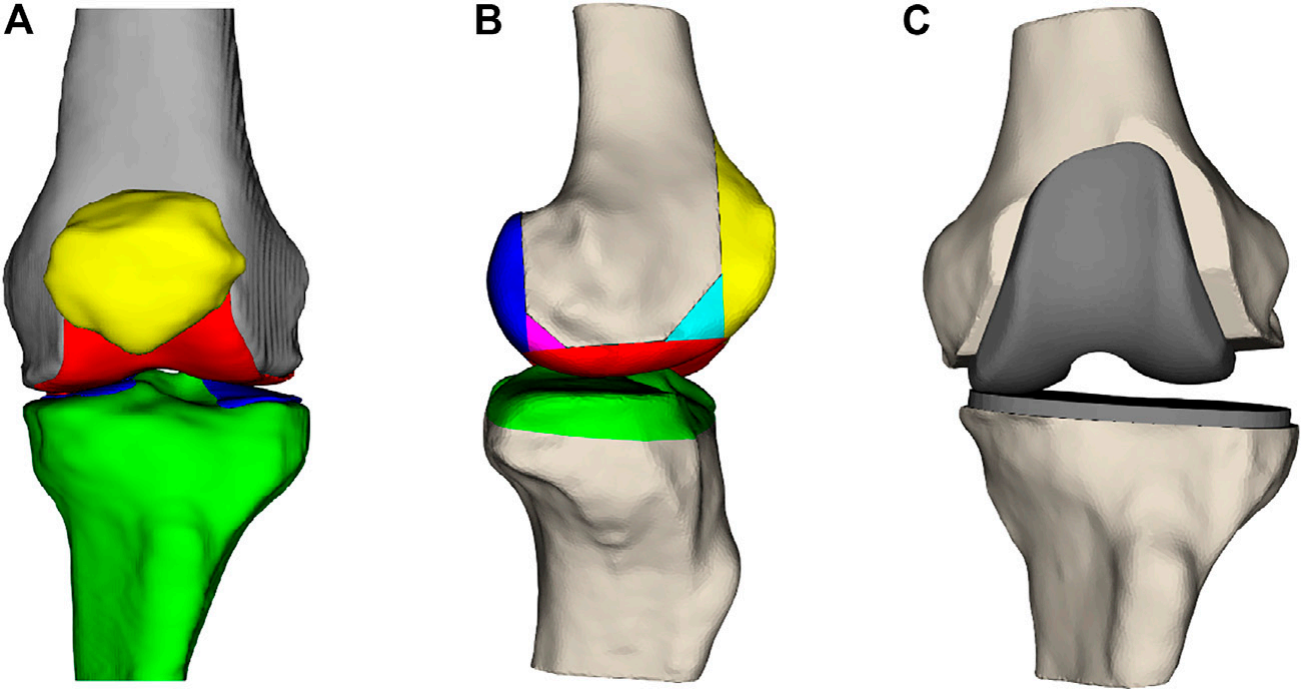
**(A)** Frontal view of the femur (gray), the femoral cartilage layer (red), the patella (yellow), the tibia (green), and the tibial cartilage (blue). **(B)** Lateral view of the femur and tibia with the parts that are resected during surgery. **(C)** Knee joint where the anterior, posterior, and distal femoral surfaces and the proximal tibial surface have been resected and replaced by a femoral and tibial implant.

Total knee arthroplasty (TKA) is a frequently performed type of surgery to improve pain symptoms, joint instability, and range of motion for patients with advanced knee arthritis (Rodríguez-Merchán and Oussedik, 2015). The knee joint comprises the femur, tibia, and patella (Figure 1A). During the TKA procedure, the femur and tibia are resected at the joint interface and resurfaced using metal implants (Figure 1B,C). Whether the patella gets resurfaced is dependent on the surgeon’s preference for the patient but is not necessary for TKA surgery to be successful (Abdel et al., 2014). There are 14 degrees of freedom to place the femoral and tibial implant components, listed in Table 1. How the surgeon positions the implants along these degrees of freedom is dependent on their surgical preferences for knee joint alignment (Cherian et al., 2014). There exist three main ways to align a knee joint during knee replacement surgery: mechanical alignment, anatomical alignment, and kinematic alignment (Cherian et al., 2014). In the end, surgeons have many possibilities to realign the knee joint, but there is no agreement on the optimal implant position (Gromov et al., 2014). This disagreement is mainly caused by inconclusive evidence on which alignment strategy results in the most optimal long-term patient outcome (Cherian et al., 2014).

**TABLE 1:** The seven transformations for the femoral and tibial implants, resulting in 14 degrees of freedom.

Implant degrees of freedom		
Transformation (units)	Femur	Tibia
Size change (size)	Implant size	Implant size
Coronal rotation (degrees)	Varus/valgus angle	Varus/valgus angle
Coronal translation (millimeters)	Posterior resection	Anterior/posterior displacement
Axial rotation (degrees)	Internal/external rotation	Internal/external rotation
Axial translation (millimeters)	Distal resection depth	Proximal resection depth
Sagittal rotation (degrees)	Flexion/extension angle	Posterior slope
Sagittal translation (millimeters)	Medial/lateral displacement	Medial/lateral displacement

Surgeons often plan the TKA procedure in advance because it has numerous benefits (Tanzer and Makhdom, 2016). Through preoperative planning, the implant component sizes can be estimated, which allows the hospital logistics to be optimized by reducing the implant and instrumentation stock and sterilization cost (Hafez and Moholkar, 2017). In addition to these advantages, the surgeons are better prepared for the surgery to avoid unforeseen intra-operative challenges and possibly reduce the surgical time (Rodrigues and Gutierres, 2016). For 3D planning, a computed tomographic scan or magnetic resonance image (MRI) of the patient’s knee, hip, and ankle are used to create a 3D model of the patient’s knee joint by implant or instrumentation manufacturers. Based on this 3D model, a default preoperative plan is created based on some fixed surgical preferences. This plan is called the manufacturer’s preoperative plan (MPP). The surgeon can modify all 14 degrees of freedom in the MPP to fine-tune the preoperative plan in a patient-specific manner. This plan is referred to as the surgeon corrected preoperative plan (SCP). The 3D preoperative plan can be transferred to the operating room using patient-specific instrumentation (PSI), navigation systems, augmented reality, or robotic-assisted surgery.

One of the current shortcomings in the use of preoperative planning is the need for revision of MPPs by the surgeon. Okada et al. analyzed the preoperative plans for 45 TKA surgeries and found that 91.1% of cases required changes by the surgeon (Okada et al., 2017). This can be attributed to the diversity in surgical planning strategies. Because the optimal alignment strategy is unknown, different surgeons have different opinions on the optimal knee alignment (Cherian et al., 2014). Currently, MPPs are created by a fixed algorithm that applies some fixed surgical preferences. However, these algorithms cannot capture the needs of all surgeons in a patient-specific manner. A retrospective study by Schotanus et al. showed that SCPs predicted the intra-operative implant sizes correctly in more than 90% of cases while the MPPs were correct in about 80% of cases compared to the implanted component sizes (Schotanus et al., 2017). Therefore, we can conclude that preoperative planning after surgeon corrections allows for correct implant size prediction in the vast majority of cases. Pietsch et al. concluded from a prospective study on 50 cases that surgeons should not blindly accept the MPP because it would require significantly more intra-operative changes (Pietsch et al., 2013). Other prospective studies resulted in the same conclusion (Stronach et al., 2013; Cucchi et al., 2018). Furthermore, Pietsch et al. measured the average time taken by the surgeon to correct the MPP, which was found to be 8 min (Pietsch et al., 2013).

This study aimed to develop an artificial intelligence (AI) driven patient-specific planning algorithm that incorporates surgeon preferences by learning from previous cases to reduce the number of modifications surgeons need to make to the surgical plan. This could reduce the planning time required by the surgeon to plan a case and, as a result, help reduce the cost associated with preoperative planning. Machine learning has not yet been applied in the literature to create preoperative plans for joint reconstruction surgery. We hypothesize that a learning-based approach allows capturing the surgeons’ surgical preferences and applying them in a patient-specific manner.

## 2 Materials and Methods

### 2.1 Data Preprocessing

To create a machine learning model, we rely on a dataset to determine model parameters. The dataset used was retrospectively collected from 5,409 primary TKA surgeries performed by thirty-nine experienced surgeons from 38 hospitals. All surgeries were performed consecutively between September 2019 and October 2020. The patients were implanted with a Vanguard, Persona, or NexGen implant (Zimmer Biomet, Warsaw, Indiana, United States), depending on the surgeon’s preference. For each case, the MPP and SCP were collected in combination with their respective 3D bone and cartilage models derived from the MRI scans. The SCP was obtained by the treating surgeon after making his corrections to the MPP. An overview of the number of cases handled by each surgeon and their respective implant choices can be found in the Supplementary Material.

**FIGURE 2:**
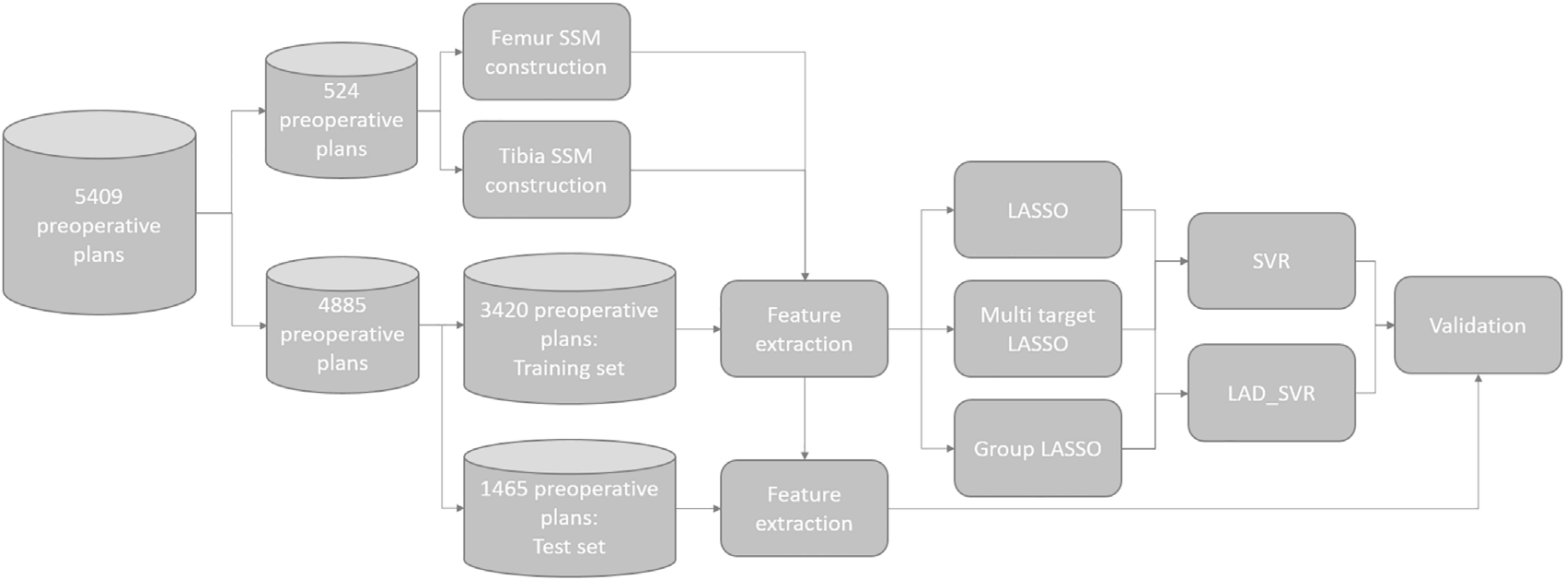
A flowchart of the dataset used for training and validation of the proposed models.

For each surgeon, the dataset was randomly split into two parts: 70% of the cases of each surgeon were used as a cross-validation dataset for creating the model and the remaining 30% of cases were used as the test set for validating the model performance. The entire data processing pipeline is illustrated in Figure 2. The goal of the machine learning model was to predict the degrees of freedom (DOFs) of the SCP (Table 1), determining the surgical plan in a surgeon-specific manner. Hence, for each of the surgeons, separate models were created using only the cases from this surgeon.

**FIGURE 3:**
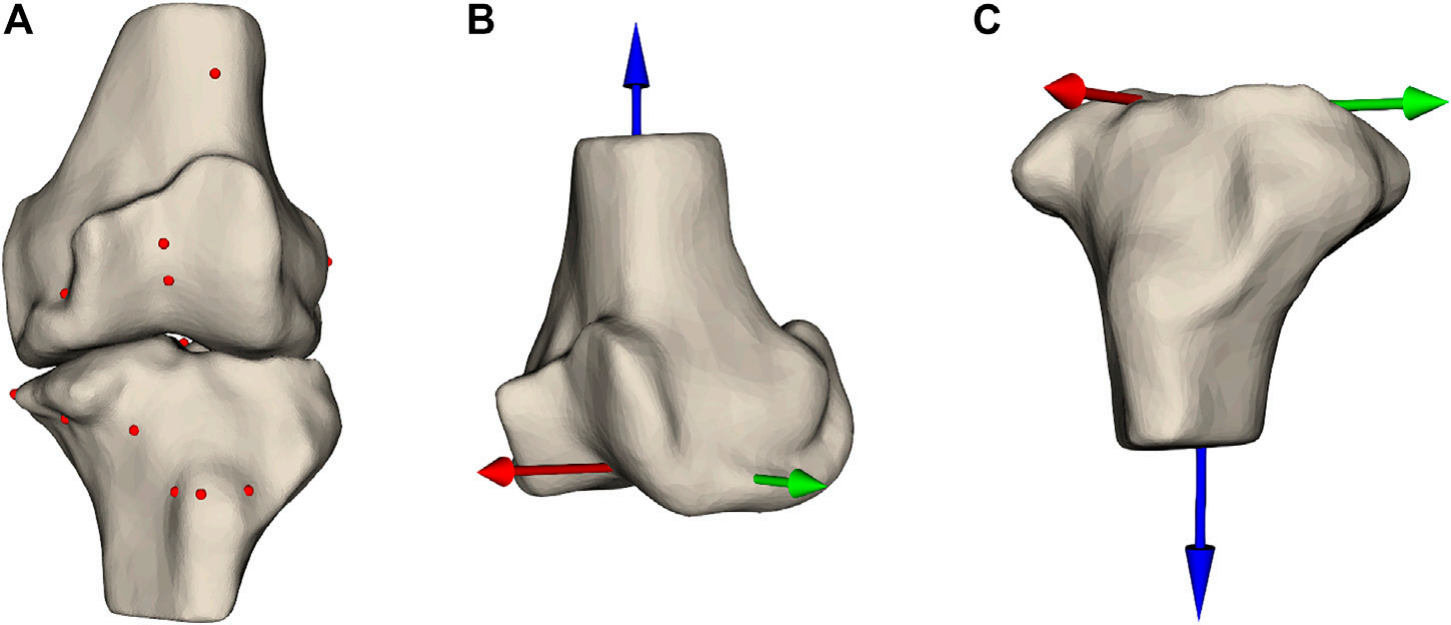
**(A)** Femur and tibia with the landmark points annotated as spheres indicating prominent structures on the bones. Panels **(B,C)** show the femoral and tibial coordinate system, respectively.

To predict these DOFs, we rely on features extracted from the MPPs. These features are descriptors of the patient’s anatomy that should enable the model to accurately predict the DOFs encompassing the SCP. The features are hand-crafted and subdivided into multiple categories: landmark locations, measurements, the DOFs in the MPPs, and shape parameters. Landmarks are prominently recognizable points that can be robustly annotated even in the presence of joint degradation. In total, 26 landmark points are used (Figure 3A, Supplementary Material). These points are chosen to allow the model to get information on the dimensions of both bones, thus allowing the implant sizes to be learned by the model. Furthermore, they serve as references for the resection levels and the rotation references. The landmark locations are expressed in two anatomical coordinate systems: the femoral coordinate system (Figure 3B) and the tibial coordinate system (Figure 3C).

**FIGURE 4:**
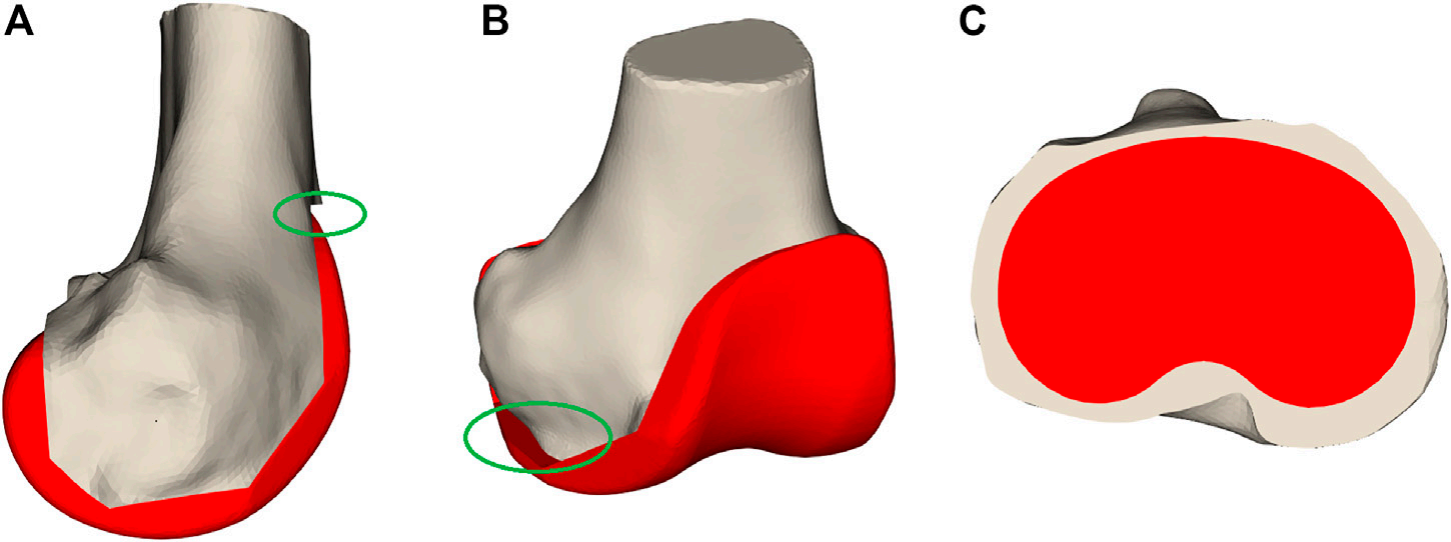
Bone measurements. Panel **(A)** displays notching where the femoral implant is too much rotated in an extended orientation that it undercuts the bones as visualized in the green oval. In **(B),** the femoral implant overhangs on the medial side indicated by the green oval. Panel **(C)** demonstrates tibial implant underhang where a large part of the tibial plateau is not covered by the implant component.

The second category of features are the measurements taken after initial virtual implantation. These are useful for indicating whether the implant components’ position and orientation should be changed. The first important measurement is femoral notching. This occurs if the femoral implant is placed too much in extension, causing the most superior tip of the implant to undercut the bone (Figure 4). Notching the femur increases the risk of post-operative bone fracture due to the stresses occurring at the interface of the implant and bone (Lesh et al., 2000). A second measurement is mediolateral femoral implant overhang, where the implant protrudes from the resected bone surface, which can cause post-operative irritation of the soft-tissue structures and thus should be avoided (Figure 4). On the tibial side, both underhang and overhang should be avoided (Figure 4). Underhang causes bone resorption resulting in implant loosening, while overhang causes soft-tissue irritation (Figure 4) (Gu et al., 2019). In total, 57 measurements have been defined (Supplementary Material).

The DOFs in the MPPs were also used as features because they provide a baseline on which the model needs to learn the necessary changes. The final set of features is shape coefficients obtained after fitting a statistical shape model (SSM) to the bones. An SSM describes the distribution of anatomical variation in a population of geometrical shapes (Cootes et al., 1995). The SSM describes a new bone as the average bone shape from the population together with a linear combination of the shape variation modes. The SSM was created based on a dataset of 524 3D models of femur and tibia (Van Dijck et al., 2018). The first fifteen shape coefficients of both femur and tibia, explaining most of the shape variation, are included as features.

### 2.2 Feature Selection

Combining all features results in a set of *d* (149) features for each surgical plan. These features for the N cases are stacked in a feature matrix $X\in R^{N\times d}$ , while the *T* DOF we want to predict are grouped in $Y\in R^{N\times T}$ . Many of these features might contain redundant or irrelevant information for the model. To obtain a subset of relevant features, multiple feature selection methods were compared. Since the problem of predicting a preoperative plan is a multi-target regression problem, we also search for a predictive subset of features for each target. Feature selection methods can be broadly categorized in three groups: filter, wrapper, and embedded approaches Bachu and Anuradha (2019). Filter methods are computationally inexpensive but result in a set of correlated features. Wrapper methods can capture these correlations and usually result in a sparser set of features compared to filter methods. However, this extra performance comes at the cost of computational time. The final class is embedded methods, which embed the feature selection step in the learning algorithm combing the best of both worlds. They can result in more optimal subsets of features compared to filter methods while being less computationally expensive than the wrapper methods. We opted to investigate the performance of three embedded feature selection methods.

The first option would be to learn one set of features predictive for all the 14 DOFs using the Multi-Task Lasso (MTL). This could be done by employing *l*_2,1_ regularization on a multi-target linear regression problem (Eq. 1) (Xiao et al. 2012). The *l*_2,1_ norm (Eq. 2) induces sparsity on the rows of the coefficient matrix Θ resulting in rows with all zeros for the features which are unpredictive of the targets. The non-zero rows of Θ indicate the features which are predictive for all targets:

$$\underset{\Theta }{\min}\;\frac{1}{2}\|Y-X\Theta {\|}_{2}^{2}+\lambda \|\Theta {\|}_{2,1},$$ (1)

$$\|A{\|}_{2,1}=\underset{i}{\sum }\sqrt{\underset{j}{\sum }A_{ij}^{2}},$$ (2)

A second method is the least absolute shrinkage and selection operator (Lasso), which induces sparsity on a set of coefficients from a linear regression model for each individual target Tibshirani (1996). This could possibly be advantageous as each DOF will be influenced by its own subset of features. The Lasso coefficients are generated for all *T* DOF of the preoperative plan (Eq. 3). The coefficients of *θ*_*t*_ which are non-zero correspond to the selected features for the corresponding target **y**_**t**_:

$$\underset{\theta_{t}}{\min}\;\frac{1}{2}\|y_{t}-X\theta_{t}{\|}_{2}^{2}+\lambda \|\theta_{t}{\|}_{1}\;t=1,2,\ldots ,T$$ . (3)

The final feature selection method is the group Lasso (Eq. 4) Yuan and Lin (2006). The group Lasso induces sparsity in groups of features. These groups are manually defined prior to fitting the model. We opted for this method because the landmark coordinates show a clear grouping structure, where the *x*, *y*, and *z* coordinates are grouped together. For that reason, all landmark features are grouped, which is indicated by the corresponding set of model parameters $\theta_{t}^{g}\in R^{3}$ . The other features form a group by themselves $\theta_{t}^{g}\in R^{1}$ . The group sparsity is induced by calculating the sum of all *l*_2_ norms of the group coefficient vectors. This will set the coefficients of some of the groups to zero, meaning they are unimportant to the model. Using the group Lasso, all coordinates of a landmark are always selected together, which is an advantage over the classical Lasso algorithm. To account for the difference in group size, a correction factor equal to the square root of the group size *σ*_*g*_ is introduced (Hastie et al., 2015):

$$\underset{\theta_{t}}{\min}\;\frac{1}{2}\|y_{t}-X\theta_{t}{\|}_{2}^{2}+\lambda \underset{g\in G}{\sum }\sqrt{\sigma_{g}}\|\theta_{t}^{g}{\|}_{2}\;t=1,2,\ldots ,T.$$ (4)

The optimal value for *λ* in each of the three feature selection methods was obtained through tenfold cross-validation on the regularization path on the training set.

### 2.3 Regression Analysis

The feature subsets selected by the algorithms from Section 2.2 are used as input to a regression framework to predict the DOFs in the SCP. All DOFs indicating the position and orientation are adaptable by increments of 0.5 mm and 0.5°, respectively. The different implant sizes are coded as ordinal variables. Predicting implant sizes could be seen as a classification problem. However, the largest and smallest sizes are implanted very infrequently. Hence, there is a large class imbalance. Because all DOFs are either ordinal or continuous data, we decided to predict all DOFs using regression models. Two methods for the regression framework were compared: support vector regression (SVR) and least absolute deviation support vector machines (LAD-SVR).

The support vector regression proposed by Vapnik tries to fit a non-linear function through a set of points (Vapnik et al., 1996). Support vector machines are universal approximators (Hammer and Gersmann, 2003). Thus, their hypothesis space can approximately model any target function. To model non-linearities, we applied a Gaussian radial basis function (RBF) kernel to the Lagrange dual formulation.

The SVR method has two downsides: the large number of inequalities slowing down the optimization and the large number of hyper-parameters (*ϵ*, C, *σ*) resulting in a long optimization time. These two problems have been solved by the least-squares support vector machine (LS-SVM), which has only one equality constraint per data sample instead of the four inequality constraints per sample of SVR (Suykens et al., 2002). The LS-SVR problem can be solved as a linear system of equations, making it much faster than the SVR. However, the downside is that the LS-SVR problem minimizes a least-squares loss sensitive to outliers. Because our dataset contains outliers, this is not desired. In 2013, Wang et al. proposed the least absolute deviation support vector regression (LAD-SVR) method, where a Huber loss is used, which is much less sensitive to outliers compared to the least-squares loss function (Wang et al., 2014). Similar to the normal support vector regression, non-linearities were modeled by RBF kernels. The LAD-SVR is optimized by the Newton method, which is numerically fast to solve for problems with a small sample size, as is the case here. As some of the surgeons make infrequent corrections to certain DOFs, a robust regression method could be beneficial.

For each of the preoperative planning parameters that need to be estimated, three SVR and three LAD-SVR models are fitted by varying the three feature subsets obtained from the different feature selection algorithms in Section 2.2. Tenfold cross-validation was used in combination with grid search to find the optimal hyper-parameter using logarithmically spaced values ∈ (10^–6^, 10^6^) and optimized independently for each DOF.

### 2.4 Statistical Analysis

The AI-based preoperative plan (APP) is defined by the best performing ML-based method during cross-validation for each individual surgeon and each planning parameter separately. A correction is counted per 0.5 mm change in resection level, 0.5°change in implant rotation, and per implant size change. These discrete steps are also applied by the surgeon when planning a real case. A Friedman test was used to compare the differences between the number of corrections required to the predictions of different machine learning models. One-sided Wilcoxon signed-rank tests will be used to test for differences between individual planning algorithms. In our experiment, we used a value of 0.01 for alpha and a power of 80%. Based on these values, a sample size calculation was done using the mean and standard deviation of the number of corrections required to the MPP (7.13 ± 4.2) and an estimate of the corrections needed for the APP (5.00 ± 2.4). This estimate relies on a 30% reduction in corrections required to the APP compared to the APP and a reduction in variance on the required number of corrections to the APP. This resulted in a sample size of 154 cases. Our test set contains 1,465 cases, surpassing the required sample size.

## 3 Results

The test set accuracies of the different ML models are compared in Table 2. The predictive performance is measured as the average number of corrections the surgeon needs to make to each of the preoperative plans generated by the different algorithms with respect to the ground truth SCP. The improvement is the percentage reduction in corrections required to the APP compared to the MPP. For 37 out of 39 surgeons, there was an improvement to the preoperative plans using machine learning to predict its planning parameters. For the two remaining surgeons, the APP and MPP were equally accurate. The methods using Lasso as feature selection were most frequently successful. The combination of Lasso with LAD-SVR was the method that most frequently resulted in the best performance. This was the case for 20 out of 39 surgeons. The model accuracy averaged over all surgeons can be found in Table 2. On average, the APP requires 3.76 corrections, while the MMP requires 7.13, a 39.71% improvement. If we average the model accuracies only for surgeons who make frequent corrections (more than three changes on average to the MPPs), the improvement increases to 47.95%.

**TABLE 2:** The number of corrections required by surgeons on average (standard deviation) to the different types of preoperative plans.

MPP	Lasso		Group Lasso		Multi-Task Lasso		APP	Improvement (%)
	SVR	LAD-SVR	SVR	LAD-SVR	SVR	LAD-SVR		
7.13 (4.93)	4.10 (2.63)	4.15 (2.62)	4.25 (2.91)	4.31 (2.87)	5.98 (4.29)	4.46 (2.87)	3.76 (2.49)	39.71% (22.89%)

The Friedman test, comparing differences between the six combinations of the three feature selection methods with the two regression methods, results in a *p*-value of 2.13e-22. For this reason, we reject the null hypothesis and use the Wilcoxon signed-rank tests to compare individual models. One-sided Wilcoxon signed-rank tests were used to identify statistically significant improvements between different preoperative planning methods. For each comparison, the *p*-values can be found in Table 3. All of the proposed machine learning algorithms were significant improvements compared to the MPP. The two methods with the Lasso as feature selection method also significantly outperformed the group Lasso and Multi-Task Lasso. In contrast, the group Lasso-based models significantly improved the Multi-Task Lasso methods. Finally, the APP significantly improved compared to using just a single machine learning method for all planning parameters.

**TABLE 3:** The *p*-values associated with one-sided Wilcoxon signed-rank tests indicating whether the preoperative planning methods in the first column are an improvement upon the preoperative planning methods in the first row. Values in bold are statistically significant improvements.

		MPP	Lasso		Group Lasso		Multi-Task Lasso		APP
			SVR	LAD-SVR	SVR	LAD-SVR	SVR	LAD-SVR	
MPP		*n/a*	1	1	1	1	1	1	1
Lasso	SVR	**1.2e-07**	*n/a*	0.76	**0.000 8**	**0.000 8**	**6.3e-07**	**5.4e-06**	1
	LAD-SVR	**1.2e-07**	0.24	*n/a*	**0.001 3**	**0.000 33**	**1.8e-06**	**5.4e-06**	1
Group Lasso	SVR	**1.2e-07**	1	1	*n/a*	0.49	**8.1e-07**	**0.001 6**	1
	LAD-SVR	**1.8e-07**	1	1	0.51	*n/a*	**6.8e-06**	**0.000 1**	1
Multi-Task Lasso	SVR	**2.7e-07**	1	1	1	1	*n/a*	1	1
	LAD-SVR	**1.8e-07**	1	1	1	1	**2e-05**	*n/a*	1
APP		**5.7e-08**	**8.4e-08**	**1.8e-07**	**1.2e-07**	**8.4e-08**	5**.7e-08**	**8.4e-08**	*n/a*

As explained in the caption of the table, these are the statistically significant improvements.

**FIGURE 5:**
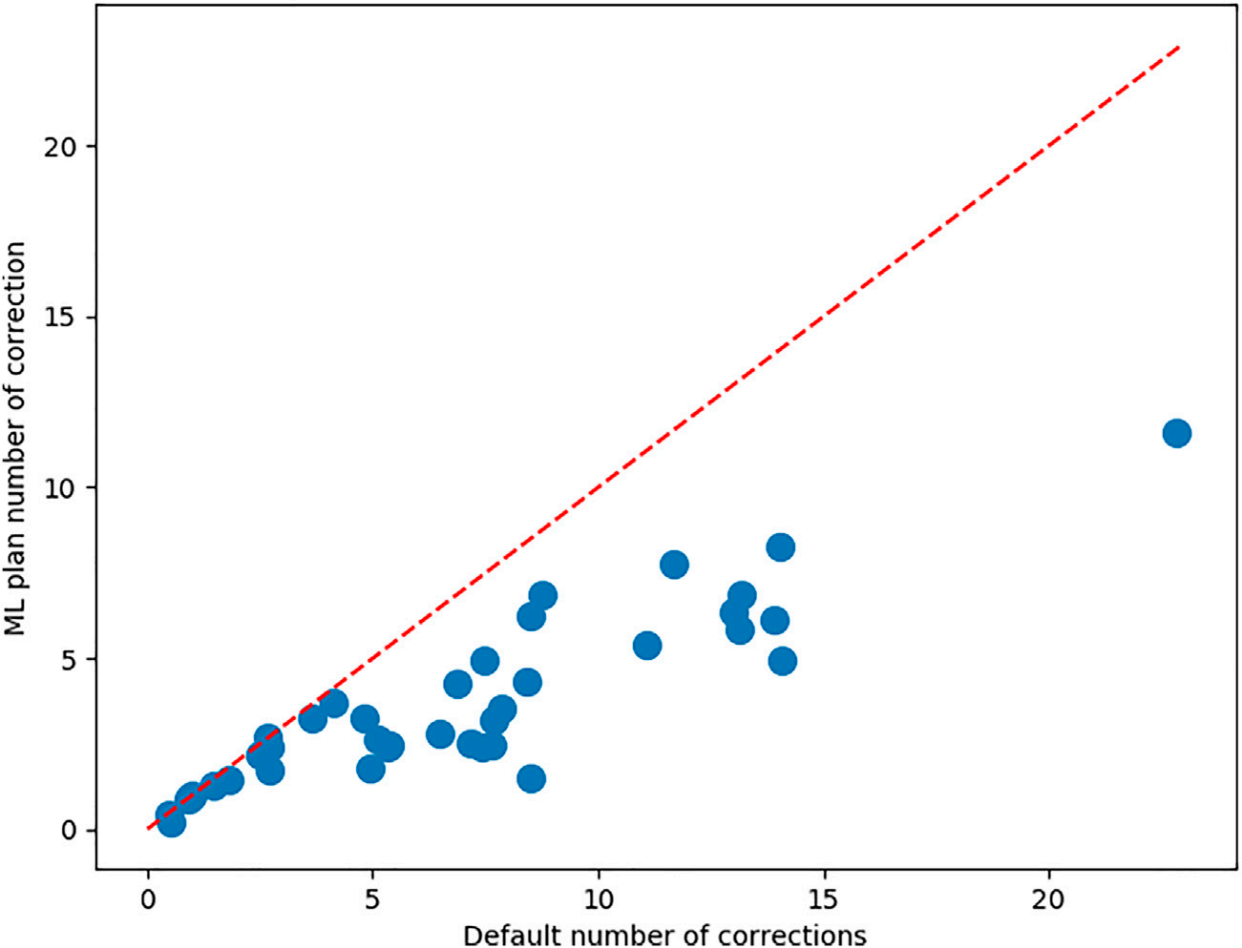
For each of the surgeons, the average number of corrections to be made to the MPP and the best ML plan. The red bisector indicates the line where no improvement occurs upon the MPP.

**FIGURE 6:**
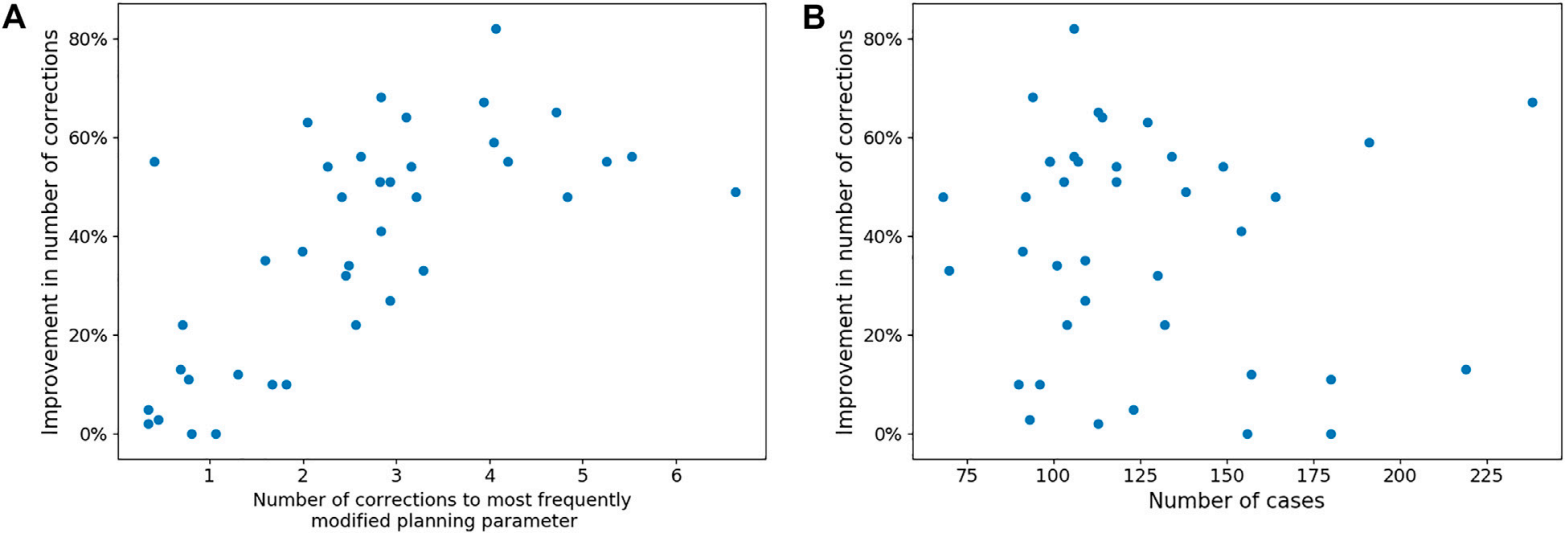
Panel **(A)** shows the trend between the number of corrections made to the most frequently modified planning parameter in the MPP of each surgeon and the reduction in corrections needed to the best ML-based plan. Panel **(B)** shows the lack of a trend between the number of cases available per surgeon and the improvement that can be made with ML-based planning.

Figure 5 shows the number of corrections each surgeon has to make to both the MPP and the best ML preoperative plan. It also shows a correlation between the number of corrections that a surgeon needs to make to the MPP and the improvement obtained using ML. The associated Pearson correlation coefficient is 0.546. From Figure 6, one can observe the correlation between average number of corrections made by the surgeon to the single planning parameter with most corrections and the improvement obtained from AI planning with Pearson correlation of 0.69. Finally, the number of cases which can be used to train the model for a surgeon is uncorrelated with the improvement caused by ML based planning, with Pearson correlation −0.095, as can be observed in Figure 6.

**FIGURE 7:**
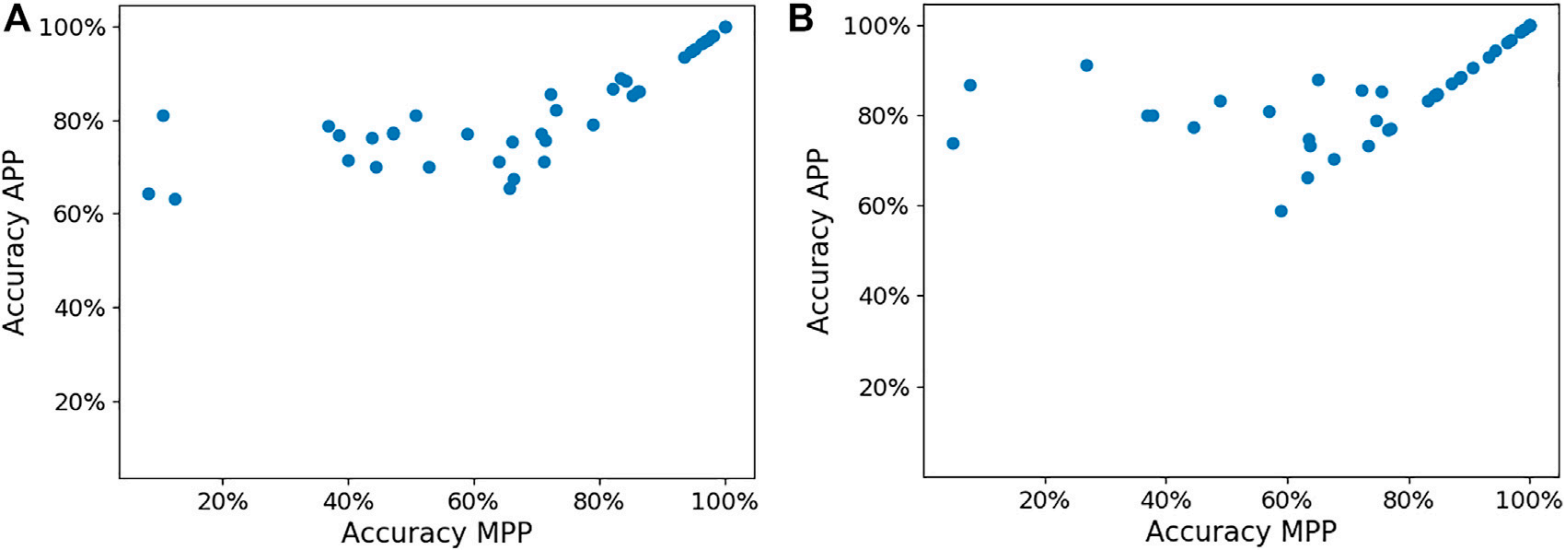
Panels **(A,B)** show the femoral and tibial implant size in the MPP and APP, respectively, for each surgeon.

The APP also helps improve the femoral and tibia implant size predictions compared to the MPP. Figure 7 presents the implant size accuracy in both MPP and APP for each surgeon. We can observe that significant accuracy improvements are possible, mainly for surgeons for whom the MPP is inaccurate. The average femoral implant size accuracy in the MPP and APP is 68.4% and 82.2% (*p*-value = 1.93e-6), respectively, while the tibial implant size accuracy in the MPP and APP is 73.1% and 85.0% (*p*-value = 2.62e-8).

## 4 Discussion

One of the benefits of applying feature selection is that we can compare the selected features with clinical knowledge to determine if the model is reliable despite being a black box. Most of the features selected can directly be related to clinical knowledge. These insights may also increase the level of trust surgeons have in the models. For example, in predicting the femoral implant size, the Lasso method found the MPP femoral implant size, MPP tibial implant size, femoral width, femoral implant overhang, and notching distance the most important features for most surgeons. These can all be logically explained. The MPP femoral implant size functions as a baseline from which the models predict the correction. The MPP tibial implant size is highly correlated with the femoral implant size. The femoral width and overhang are measures that the model can use to correct the implant size in case of mediolateral over- and undersizing. Finally, the notching distance is a measure predictive of under- or oversizing in the anteroposterior direction. By describing the important features to the surgeon, we can instill trust in our models.

The Multi-Task Lasso was selected as the best feature selection model in combination with either regression model for only five surgeons. The disadvantage of the Multi-Task Lasso is that it selects one set of features predictive for all planning parameters. Therefore, some of the features which are predictive of only a limited set of planning parameters might not be included in the model. The Lasso and group Lasso methods do not suffer from this effect. Therefore, they are more frequently optimal. The group Lasso is the best feature selection model for nine surgeons, while the lasso is optimal for 31 surgeons. We attribute this to the fact that the group Lasso has to include all three coordinates of the landmark locations in the model facilitating interpretability. However, this seems to come at the cost of reduced model accuracies. One disadvantage common to all feature selection methods is that interactions between features are not considered. The LAD-SVR and SVR methods were most accurate for 24 and 18 surgeons, respectively. The LAD-SVR method has the advantage of being less sensitive to outliers due to the use of the Huber loss function in contrast with the *ϵ*-insensitive loss. This is beneficial for surgeons who make infrequent but large changes to a planning parameter.

Overall, machine learning can significantly improve upon measurement and rule-based systems as in the MPP. Only for two surgeons, the APP did not improve upon the MPP. These two surgeons make very few changes (1.0 and 2.68 changes, resp.) to the preoperative plan. Hence, there are only a few cases from which the machine learning models can learn, limiting the possible improvement. Nevertheless, over the entire sample of surgeons, the APP is a significant improvement over the MPP (*p*-value = 5.7e-08). For surgeons who make frequent changes ( > 3 changes) to the MPP, almost 50% fewer corrections are needed to the APP. This may help reduce the time spent on preoperative planning for TKA. The average accuracy improved and the consistency of the predicted planning parameters increased as noted from the lower standard deviation in the number of corrections that need to be made to the APP compared to the MPP. It can also be concluded that using a combination of different ML methods for different planning parameters significantly improves the overall quality of the preoperative plans compared to using a single method for all planning parameters. The most important planning parameters are the implant sizes. The APP significantly improves the implant size accuracy over the MPP. These improvements are mainly for surgeons for whom the MPP has low implant size prediction accuracy. In contrast, surgeons for whom the implant size accuracy in the MPP is above 80% do not experience any improvement from the APP.

The reduction in corrections needed by the surgeon is highly correlated with the number of corrections made to the MPPs. This result might be explained by the increased number of samples from which the model can learn how to correct the MPP to be closer to the SCP. However, this measure is not the only predictor of the improvement that can be obtained by machine learning-based preoperative planning. Because these corrections might be divided over all planning parameters, all planning parameters have small corrections. This is also problematic because the samples with small corrections to a planning parameter are also harder to learn from. Hence, the largest correlation with the machine learning-based planning improvement observed was the maximum over all planning parameters of the average number of corrections made to it. If a surgeon makes large changes to a specific planning parameter from the MPP, then the trend can more easily be learned by the machine learning model. Surprisingly, the number of cases planned by the surgeon was uncorrelated with the improvement that can be made by machine learning-based preoperative plans. This is a counterintuitive finding because, in general, machine learning models perform better with more data to train the model. Having more training data only helps if the surgeons make corrections that are consistent and frequent. Therefore, an ideal dataset size that generally results in accurate AI-generated preoperative plans cannot be easily proposed. Nevertheless, every surgeon in our dataset had at least 75 cases, which is a large dataset. Therefore, our modeling approach is limited to high-volume surgeons. One possible topic of future research could be to investigate online learning to predict preoperative plans. This allows the models to improve over time as the surgeon plans more cases. With advances in basic and clinical science, surgeons’ approach to TKA also evolves over time. Therefore, the second benefit of online learning is that it allows the AI plans to evolve over time with changes in the surgeon’s planning method.

The main limitation of our approach is that it relies on static bone-derived features. Even though bone anatomy is very important to determine implant function, the soft-tissue structures also play an important role. They dictate the stability and aid in the motion of the joint. Currently, our models do not capture the dynamic nature of the arthritic knee because they are solely based on an MRI scan. Therefore, we hypothesize that adding these dynamic measures would help improve the quality of the models. Currently, robotics is starting to be increasingly used in TKA. Most of these robotic systems allow the measurement of knee dynamics (Keggi et al., 2021). These measures of soft-tissue balance throughout the range of motion could be included as features. Unfortunately, the adoption of robotic-based TKA is still limited to high-volume surgical centers. Therefore, another option could be to rely on musculoskeletal modeling to simulate knee kinematics and ligament elongation (Vanheule et al., 2016; Bartsoen et al., 2021). Using these models, a knee squat motion could be simulated, subsequently extracting features from them. At the time of our study, these dynamic knee motion-derived features were not available. Hence, further research is required to investigate if they help improve model accuracy.

To the best of our knowledge, no literature predicted preoperative plans for TKA or orthopedic surgery using machine learning. Nevertheless, several studies have attempted to predict the implant sizes used intraoperatively. Most notably, Kunze et al. compared support vector machines, stochastic gradient boosting, elastic net penalized linear regression, and random forests and extreme gradient boosting to predict femoral and tibial implant sizes (Kunze et al., 2022). They relied on patient age, gender, height, weight, and body mass index to predict the component sizes. For their study a cohort of 11,777 cases was collected by 21 surgeons all using the same implant type. Optimal results were obtained using support vector machines for predicting the femoral implant size with an accuracy of 42.2%. For the tibial implant size, elastic net penalized linear regression was optimal with 43.8% accuracy. Wallace et al.’s study found similar results for predicting TKA intraoperative implant sizes based on demographic data (Wallace et al., 2020). Using linear regression based on patient age, gender, height, weight, and race the predicted component sizes with accuracy of 43.7% for femoral and 43.7% for tibial implant size. Although their studies are not directly comparable to ours due to the difference of the preoperative and intraoperative setting, our method yields significantly higher accuracy. We mainly attribute these accuracy differences to the extra information, which can be obtained from anatomical measurements compared to the simpler demographic data from the study of Kunze and Wallace.

One of the factors affecting the performance of machine learning-based preoperative plans is the quality of the data used to train the models. One problem is the definition of a correct preoperative plan. Currently, there is a large variation in knee alignment methods that surgeons use, such as mechanical, anatomical, kinematic, restricted kinematic, adjusted mechanical, inverse kinematic, and functional alignment (Howell et al., 2013; Winnock de Grave et al., 2020; Vendittoli et al., 2021; Kayani et al., 2020; Cherian et al., 2014). These different alignment methods exist because surgeons do not agree on the alignment method, which results in optimal long-term patient outcomes (Rivière et al., 2017). This disagreement results from lacking data and large-scale studies comparing the patient outcome of different knee alignment methods. Therefore, having a consensus on the values of preoperative planning parameters amongst surgeons will not be possible because they might strive for different targets. The definition of a “correct” preoperative plan is hence the topic of further clinical research. As a result, our ground truth data are obtained each time by the single surgeon who executed the procedure. A second factor affecting data quality is the intra-observer variability of a surgeon while planning. Schoenmakers et al. investigated the intra-observer variability of MRI-based preoperative planning based on the intraclass correlation coefficient (ICC) of repeated planning of 40 cases by five surgeons (Schoenmakers et al., 2021). They found that, for two and three planning parameters, the ICC was moderate (0.5 < ICC ≤0.75) and poor (ICC ≤0.5), respectively. Both the inter-surgeon and intra-surgeon variability in preoperative planning imposes a limitation on the quality of the data used in our study.

Several methods could be proposed to remove cases with low-quality data. First of all, the post-operative patient-reported outcome scores allow the removal of cases with bad outcomes. This limits the presence of low-quality preoperative plans in the training set. Consequently, the machine learning models are less likely to predict thee preoperative plans resulting in sub-optimal surgical outcomes. Secondly, the cases in which the surgeon deviates significantly from the preoperative plan during the surgery should also be removed from the dataset because the preoperative plans were not correct. Lastly, the data quality can be improved by asking the surgeons to plan each case multiple times, which allows for the removal of cases with large variability in implant position, orientation, or size.

Besides improvements in data quality, the machine learning models could be further improved. Our feature selection methods only consider the main effects while neglecting interactions between features. Secondly, our method relies on manually defined features based on clinical knowledge. However, as different surgeons consider different anatomical and kinematic parameters, some features might not be captured by our model. Therefore, one of our future goals is to use the entire 3D bone model, which could mathematically be represented by a graph to be used as input to a machine learning model. This would reduce the need for manually defining clinical features because they could be learned from a dataset of 3D bone models, for example, by using graph convolutional neural networks (Wu et al., 2019).

## 5 Conclusion

This study aimed to evaluate whether machine learning can be applied to improve the default preoperative plans for TKA provided by instrumentation manufacturers to a surgeon. A machine learning-based preoperative plan, which captures surgical preferences in a patient- and surgeon-specific manner, has the potential to reduce the time needed to modify the preoperative plan prior to approval. Our method that used hand-crafted features based on clinical knowledge combined with sparsity-inducing algorithms for feature selection and non-linear regression was able to reduce the average amount of corrections needed by surgeons by 39.71%.

## Data Availability

The dataset presented in this article is not readily available because of the contractual limitation imposed upon Materialise with regard to data sharing. Requests to access the dataset should be directed to AL, adriaan.lambrechts@materialise.be.
