# Corrigendum: Artificial Intelligence Based Patient-Specific Preoperative Planning Algorithm for Total Knee Arthroplasty

**DOI:** 10.3389/frobt.2022.899349

**Published:** 2022-04-28

**Authors:** Adriaan Lambrechts, Roel Wirix-Speetjens, Frederik Maes, Sabine Van Huffel

**Affiliations:** ^1^ Materialise NV, Leuven, Belgium; ^2^ Department of Electrical Engineering (ESAT), STADIUS Center for Dynamical Systems, Signal Processing and Data Analytics, KU Leuven, Leuven, Belgium; ^3^ Department of Electrical Engineering (ESAT), Processing Speech and Images (PSI), KU Leuven, Leuven, Belgium; ^4^ Medical Imaging Research Center, UZ Leuven, Leuven, Belgium

**Keywords:** total knee arthroplasty, patient-specific, preoperative planning, machine learning, orthopedic surgery, artificial intelligence

In the original article, the “Statistical shape model-based prediction of tibiofemoral cartilage” was not cited. The citation has now been inserted in the section **Materials and Methods**, “Data Preprocessing,” Paragraph 5 and should read:

“The DOFs in the MPPs were also used as features because they provide a baseline on which the model needs to learn the necessary changes. The final set of features is shape coefficients obtained after fitting a statistical shape model (SSM) to the bones. An SSM describes the distribution of anatomical variation in a population of geometrical shapes (**Cootes et al., 1995**). The SSM describes a new bone as the average bone shape from the population together with a linear combination of the shape variation modes. The SSM was created based on a dataset of 524 3D models of femur and tibia (Van Dijck et al., 2018). The first fifteen shape coefficients of both femur and tibia, explaining most of the shape variation, are included as features.”

An **Acknowledgements** section was also not included in the published article. A corrected statement appears below.

“The authors gratefully acknowledge Christophe Van Dijck for the construction of the knee statistical shape models.”

The authors apologize for these errors and state that this does not change the scientific conclusions of the article in any way. The original article has been updated.
