# Supplementary material for: Artificial Intelligence Based Patient-Specific Preoperative Planning Algorithm for Total Knee Arthroplasty
Source: Front Robot AI. 2022 Mar 8;9:840282. doi: 10.3389/frobt.2022.840282 (PMC8957999; doi:10.3389/frobt.2022.840282)
Supplement: Supplementary file 1 [file DataSheet1.pdf]

Supplementary Material

1 DATA DESCRIPTION

Table S1. An overview of the number of cases for each surgeon and the implant brand they use.

Surgeon ID	Number of cases	Implant system
Surgeon 1	75	Persona
Surgeon 2	76	Persona
Surgeon 3	92	NexGen
Surgeon 4	93	Vanguard
Surgeon 5	94	Persona
Surgeon 6	96	Persona
Surgeon 7	99	Persona
Surgeon 8	99	Vanguard
Surgeon 9	101	Vanguard
Surgeon 10	102	Persona
Surgeon 11	103	Persona
Surgeon 12	104	Vanguard
Surgeon 13	104	Persona
Surgeon 14	104	Vanguard
Surgeon 15	105	Persona
Surgeon 16	106	Vanguard
Surgeon 17	108	Vanguard
Surgeon 18	108	Vanguard
Surgeon 19	110	Persona
Surgeon 20	112	Vanguard
Surgeon 21	115	Vanguard
Surgeon 22	115	Vanguard
Surgeon 23	121	Vanguard
Surgeon 24	123	Persona
Surgeon 25	127	Persona
Surgeon 26	129	Persona
Surgeon 27	131	Vanguard
Surgeon 28	133	Vanguard
Surgeon 29	142	Vanguard
Surgeon 30	149	Vanguard
Surgeon 31	151	Persona
Surgeon 32	152	Vanguard
Surgeon 33	155	Persona
Surgeon 34	160	Persona
Surgeon 35	176	Vanguard
Surgeon 36	176	Vanguard
Surgeon 37	180	Vanguard
Surgeon 38	218	Persona
Surgeon 39	237	Vanguard

2 FEATURE DESCRIPTION

Table S2. List of all landmark points with their description and the coordinate system in which they are defined.

Landmark name	Description	Coordinate system
pt_notch	Most anterior point on the femur at the height of the superior tip of the femoral implant's anterior flange	Femoral coordinate system
pt_piri_fossa	Most medial point of the piriformis fossa of the femur	Femoral coordinate system
pt_fem_head	Center point of the femoral head	Femoral coordinate system
pt_dislat_cart	Most distal point on the cartillage of the lateral femoral epicondyle	Femoral coordinate system
pt_dismed_cart	Most distal point on the cartillage of the medial femoral epicondyle	Femoral coordinate system
pt_poslat	Most posterior point on the lateral epicondyle of the femoral bone	Femoral coordinate system
pt_poslat_cart	Most posterior point on the cartillage of the lateral epicondyle of the femur	Femoral coordinate system
pt_posmed	Most posterior point on the medial epicondyle of the femoral bone	Femoral coordinate system
pt_posmed_cart	Most posterior point on the cartillage of the medial epicondyle of the femur	Femoral coordinate system
pt_trochlea	The most distal point on the femoral trochlea region	Femoral coordinate system
pt_trochlea_cart	The most distal point on the femoral cartilage trochlea region	Femoral coordinate system
pt_lat_tub	The lateral most point on the superior edge of the tibial tuberosity	Tibial coordinate system
pt_med_tub	The medial most point on the superior edge of the tibial tuberosity	Tibial coordinate system
pt_malleolus	The center point between the medial and lateral malleolus at the superior tip of the talus	Tibial coordinate system
pt_lat_spine	The most superior point of the lateral spine point of the tibia	Tibial coordinate system
pt_med_spine	The most superior point of the medial spine point of the tibia	Tibial coordinate system
pt_med_tibia	Most medial point on the tibial plateau	Tibial coordinate system
pt_med_pcl	Most medial point of the posterior cruciate ligmant insertion region on the tibial plateau	Tibial coordinate system
pt_lat_pcl	Most lateral point of the posterior cruciate ligmant insertion region on the tibial plateau	Tibial coordinate system
pt_canal	Center of the central point of the intramedullary canal of the tibia proximal to the tibial tuberosity	Tibial coordinate system
pt_spine	The center point between the medial and laterl tibial spine points	Tibial coordinate system
pt_lat_prox_cart	The most proximal point on the cartilage of the lateral tibial plateau	Tibial coordinate system
pt_med_prox_cart	The most proximal point on the cartilage of the medial tibial plateau	Tibial coordinate system
pt_fib	The most proximal point of the fibula	Tibial coordinate system
pt_lat_plateau	The most distal point on the cartilage of the lateral tibial plateau	Tibial coordinate system
pt_med_plateau	The most distal point on the cartilage of the medial tibial plateau	Tibial coordinate system

Table S3. A list of all measurements and their description which are used as features in the model.

Measurement	Description
Femur implant size	Femoral implant size in the MPP
Femoral distal resection	Femoral distal resection depth in the MPP
Femoral posterior resection	Femoral posterior resection depth in the MPP
Femoral implant flexion/extension angle	Femoral implant flexion/extension rotation angle in the MPP
Femoral implant internal/external rotation	Femoral implant internal/external rotation angle in the MPP
Femoral implant varus/valgus angle	Femoral implant varus/valgus rotation angle in the MPP
Tibial implant size	Tibial implant size in the MPP
Tibial proximal resection	Tibial proximal resection level in the MPP
Tibial implant posterior slope	Tibial implant posterior slope in the MPP
Tibial implant internal/external rotation	Tibial implant internal/external rotation angle in the MPP
Tibial implant varus/valgus angle	Tibial implant varus/valgus rotation angle in the MPP
Leg side	Side of the leg being operated on (left or right)
Notching distance	To distance from the superior tip of the anterior femoral implant flange to the femoral bone
Femoral width	The distance between the MCL and LCL attachment points on the femur
posterior - AP axes angle	Angle between the femoral anterioposterior axis and the posterior axis in the distal cut plane defined in the MPP
epicondylar-posterior angle	Angle between the epicondylar axis and the posterior cut plane defined in the MPP
Distal lateral resection depth	Distal resection depth on the lateral femoral epicondyle including the cartilage layer, measured in the MPP
Distal medial resection depth	Distal resection depth on the medial femoral epicondyle including the cartilage layer, measured in the MPP
epicodylar axis length	Distance between the medial and lateral epicondyle points
Posterior lateral resection depth	Posterior resection depth on the lateral femoral epicondyle including the cartilage layer, measured in the MPP
Posterior medial resection depth	Posterior resection depth on the medial femoral epicondyle including the cartilage layer, measured in the MPP
Hip knee ankle angle	Angle between the mechanical axes of the femur and tibia
Proximal lateral resection depth	The resection depth on the proximal tibia including cartilage measured on the lateral side, measured in the MPP
Proximal medial resection depth	The resection depth on the proximal tibia including cartilage measured on the medial side, measured in the MPP
Femoral valgus angle	The angle between the femoral mechanical and anatomical axis
Femoral implant overhang	Femoral implant overhang measured as the difference between the width of the femoral implant and the width of the femoral distal cut surface
Tibial implant mediolateral overhang	Tibial mediolateral implant overhang measured as the difference between the width of the tibial implant and the width of the tibial proximal cut surface
Tibial implant anteroposterior overhang	Tibial anteroposterior implant overhang measured as the difference between the length of the tibial implant and the maximal length of the tibial proximal cut surface
Femur SSM Coefficients 1 - 15	The first 15 femoral shape coefficients explaining most of the femoral shape variation.
Tibia SSM Coefficients 1 - 15	The first 15 tibial shape coefficients explaining most of the tibial shape variation.
